# Evaluation of Immersion and Spray Applications of Antimicrobial Treatments for Reduction of *Campylobacter jejuni* on Chicken Wings

**DOI:** 10.3390/foods10040903

**Published:** 2021-04-20

**Authors:** Sara V. Gonzalez, Ifigenia Geornaras, Mahesh N. Nair, Keith E. Belk

**Affiliations:** Center for Meat Safety & Quality, Department of Animal Sciences, Colorado State University, Fort Collins, CO 80523-1171, USA; sara.gonzalez@colostate.edu (S.V.G.); gina.geornaras@colostate.edu (I.G.); mahesh.narayanan_nair@colostate.edu (M.N.N.)

**Keywords:** *Campylobacter jejuni*, antimicrobials, decontamination, poultry, chicken wings, application method

## Abstract

The decontamination efficacy of antimicrobial treatments against *Campylobacter jejuni* on chicken wings was evaluated. Chicken wings surface-inoculated with *C. jejuni* (3.9 log colony-forming units [CFU]/mL) were left untreated (control) or were treated by immersion (5 s) or in a spray cabinet (4 s) with water, a sulfuric acid and sodium sulfate blend (SSS; pH 1.2), formic acid (1.5%), peroxyacetic acid (PAA; 550 ppm), or PAA (550 ppm) that was pH-adjusted (acidified) with SSS (pH 1.2) or formic acid (1.5%). All evaluated immersion and spray chemical treatments effectively (*p* < 0.05) lowered *C. jejuni* populations on chicken wings. Spray application of chemical treatments resulted in immediate pathogen reductions ranging from 0.5 to 1.2 log CFU/mL, whereas their application by immersion lowered initial pathogen levels by 1.7 to 2.2 log CFU/mL. The PAA and acidified PAA treatments were equally (*p* ≥ 0.05) effective at reducing initial *C. jejuni* populations, however, following a 24 h refrigerated (4 °C) storage period, wings treated with acidified PAA had lower (*p* < 0.05) pathogen levels than samples that had been treated with PAA that was not acidified. Findings of this study should be useful to the poultry industry in its efforts to control *Campylobacter* contamination on chicken parts.

## 1. Introduction

The Foodborne Diseases Active Surveillance Network (FoodNet) of the Centers for Disease Control and Prevention’s (CDC) Emerging Infections Program reported that in 2019, *Campylobacter* was the leading bacterial cause of foodborne illness with an incidence rate of 19.5 cases per 100,000 population [1]. Specifically, out of 25,866 total cases of foodborne illness that were laboratory-diagnosed in that year, 9731 were due to infection with *Campylobacter* [1]. Individuals with *Campylobacter* infections, however, do not always seek medical treatment and even if they do, cases may remain undiagnosed [2]. Therefore, when underreporting and underdiagnosis are factored in, estimates indicate that *Campylobacter* spp. are actually responsible for 1.5 million diarrheal illnesses each year in the United States [2,3]. The most common species of *Campylobacter* associated with human campylobacteriosis cases is *C. jejuni*, and is responsible for at least 80% of *Campylobacter* enteric infections [4,5].

*Campylobacter* infections are primarily associated with consumption of unintentionally undercooked contaminated poultry products [6,7]. Moreover, *Campylobacter* in poultry is the number one pathogen-food combination in terms of annual illness burden, with a total of 608,231 infections and an estimated cost of more than $1.2 billion [8]. In an effort to reduce the incidence of foodborne illness cases from poultry products, slaughter facilities are required by the U.S. Department of Agriculture’s (USDA) Food Safety and Inspection Service (FSIS) to identify points during slaughter and processing where physical and/or chemical interventions can be applied to reduce pathogen contamination levels [9]. In the United States, FSIS Directive 7120.1 [10] provides poultry processors with a list of antimicrobials that are approved for use as decontamination treatments of poultry products. Peroxyacetic acid (PAA), which is currently the most widely used antimicrobial intervention in U.S. poultry processing facilities, is approved for use up to a maximum concentration of 2000 ppm [10,11]. Also approved are various organic and inorganic acids, cetylpyridinium chloride, chlorine, acidified sodium chlorite, trisodium phosphate, and a blend of sulfuric acid and sodium sulfate (SSS; also referred to as AFTEC 3000 or Amplon in the literature) [10,11]. SSS can be used as a spray, immersion, or wash treatment of poultry products at concentrations that would achieve a targeted pH range of 1.0 to 2.2 [10].

Performance standards for *Salmonella* and *Campylobacter*, established by FSIS, are used to assess the effectiveness of decontamination interventions used by a facility, in limiting or reducing pathogen contamination [12]. Since more than 85% of poultry meat in the United States is sold as parts, FSIS includes in its testing program sampling sites for both pathogens in the cut-up room to test poultry parts [13,14]. The current performance standards for the maximum acceptable *Campylobacter*-positives for chicken are 15.7% of broiler carcasses, 9.6% of comminuted products, and 7.7% of parts [13,15]. Thus, the poultry industry is reevaluating current antimicrobial interventions used for pathogen control and is looking for novel decontamination treatments to apply to meet the strict regulations for poultry [14,16].

There are numerous published studies on the antimicrobial effects of various chemical treatments against *Salmonella* populations on whole chicken carcasses and parts [11,14,17,18,19]. In comparison, however, fewer research studies have reported on the effect of such treatments against *Campylobacter*, and in particular, on chicken parts. Additionally, regardless of poultry product type and pathogen, studies investigating the decontamination efficacy of chemical treatments that combine two or more modes of action are also limited. Therefore, the objectives of this study were to (i) evaluate the antimicrobial effects of SSS, formic acid, PAA, and PAA that was pH-adjusted with SSS or formic acid (hereafter referred to as “acidified PAA”), when applied to chicken wings inoculated with *C. jejuni*, and (ii) determine the antimicrobial efficacy of the treatments as a result of applying the test solutions by immersion or spraying. Additionally, the antimicrobial effects against inoculated populations were evaluated immediately after treatment application (0 h) and after 24 h of storage at 4 °C.

## 2. Materials and Methods

### 2.1. Bacterial Strains and Inoculum Preparation

The inoculum consisted of a mixture of six *C. jejuni* strains of poultry origin (Table 1). Working cultures of the strains were maintained at 4 °C on plates of Campy Cefex Agar, Modified (mCCA; Hardy Diagnostics, Santa Maria, CA, USA) that were held within anaerobic containers (AnaeroPack Rectangular Jar; Mitsubishi Gas Chemical America, New York, NY, USA) with a microaerophilic environment generating gas pack (mixture of approximately 6 to 12% O_2_ and 5 to 8% CO_2_; AnaeroPack-MicroAero sachet, Mitsubishi Gas Chemical America).

The *C. jejuni* strains were individually cultured and subcultured in 10 mL of Bolton broth (Hardy Diagnostics) incubated at 42 °C for 48 h under microaerophilic conditions (Oxoid CampyGen sachet, Thermo Scientific, Basingstoke, UK). Cultures of the six strains were then combined and centrifuged (6000× *g*, 15 min, 25 °C; Sorvall Legend X1R centrifuge, Thermo Scientific, Waltham, MA, USA). Resulting cell pellets were washed twice with 10 mL of phosphate-buffered saline (PBS; pH 7.4; Sigma-Aldrich, St. Louis, MO, USA), and the final washed cell pellet comprising all six strains was resuspended in 60 mL of PBS. This cell suspension (ca. 7 log colony-forming units [CFU]/mL concentration) was then diluted 10-fold in PBS, and the diluted inoculum (ca. 6 log CFU/mL concentration) was used to inoculate the chicken wings. The concentration of the *C. jejuni* inoculum (undiluted and diluted) was determined by plating serial dilutions onto mCCA.

### 2.2. Inoculation of Chicken Wings

Fresh (i.e., not frozen) skin-on whole chicken wings were purchased from a wholesale food distributor. Wings were stored at 2 °C and were used for the study within six days of receipt. Two trials (repetitions) of the study were conducted on two separate days. On the first day of each trial, wings were randomly assigned to a control treatment or one of six treatments to be applied by immersion or spraying. For each antimicrobial treatment and application method, six samples were placed on trays lined with ethanol-sterilized aluminum foil and were inoculated under a biological safety cabinet. A 0.1 mL (100 µL) aliquot of the diluted *C. jejuni* inoculum was deposited, with a micropipette, on one side of each wing and then spread over the entire surface with a sterile disposable spreader. After a 10 min bacterial cell attachment period, samples were turned over, with sterile forceps, and were inoculated on the second side using the same procedure. The second inoculated side was also left undisturbed for 10 min to allow for inoculum attachment. The target inoculation level was 3 to 4 log CFU/mL of wing rinsate.

### 2.3. Antimicrobial Treatment of Chicken Wings

Inoculated wings were left untreated, to serve as controls, or they were treated by immersion or a spray application with water, SSS (pH 1.2; Amplon, Zoetis, Florham Park, NJ, USA), formic acid (1.5%; BASF Corporation, Florham Park, NJ, USA), PAA (550 ppm; Actrol Max, Kroff, Pittsburgh, PA, USA), PAA (550 ppm) acidified with SSS (pH 1.2; SSS-aPAA), or PAA (550 ppm) acidified with formic acid (1.5%; FA-aPAA). The water treatment was included to determine the rinsing effect of the immersion and spray treatments. Antimicrobial treatment solutions were prepared according to the manufacturers’ instructions, and the pH of solutions was measured (Orion Star A200 Series pH meter and Orion RossUltra pH electrode, Thermo Scientific, Schaumburg, IL, USA). Average pH values of the SSS, formic acid, and PAA solutions were 1.2, 2.9, and 3.2, respectively. For the SSS-aPAA and FA-aPAA solutions, average pH values were 1.2 and 2.8, respectively. The PAA concentration was verified using a hydrogen peroxide and peracetic acid test kit (LaMotte Company, Chestertown, MD, USA).

For immersion application of the test solutions, inoculated wings were individually immersed for 5 s in 500 mL of the solution in a Whirl-Pak bag (1627 mL; Nasco, Fort Atkinson, WI, USA). A different Whirl-Pak bag and fresh, unused solution was used to immersion-treat each sample. Spray application of the water and chemical treatments was performed using a custom-built spray cabinet (Birko/Chad Equipment, Olathe, KS, USA) fitted with two 0.38 L/min FloodJet spray nozzles (Spraying Systems Co., Glendale Heights, IL, USA) positioned above the product belt. The inoculated wings were placed on a cutting board on top of the ladder-style conveyor belt of the cabinet and were sprayed with the test solution at a pressure of 69 to 83 kPa and a product contact time of 4 s.

Immersion- and spray-treated wings were placed on sterile wire racks for 5 min to allow excess solution to drip off samples before microbiological analysis or refrigerated storage. For each trial, three of the six samples per treatment were analyzed for *C. jejuni* populations following treatment application (0 h analysis), and the three remaining samples were placed in individual 710 mL Whirl-Pak bags (Nasco) and analyzed after a 24 ± 1 h storage period at 4 °C.

### 2.4. Microbiological Analysis

At each sampling time (0 h and 24 h), untreated (control) and treated samples were analyzed for *C. jejuni* populations. For microbial analysis of 0 h samples, wings were placed in a Whirl-Pak bag (710 mL) containing 150 mL of neutralizing buffered peptone water (nBPW; Acumedia-Neogen, Lansing, MI, USA) [20]. For the 24 h samples, which were already in Whirl-Pak bags, 150 mL of nBPW was aseptically poured into each bag. Sample bags containing individual wings were vertically shaken by hand with a strong downward force, 60 times, to recover cells from the wing surface. Rinsates were serially diluted (1:10) in buffered peptone water (Difco, Becton Dickinson and Company, Sparks, MD, USA) and appropriate dilutions were surface-plated, in duplicate, onto pre-warmed (42 °C) mCCA plates. Plates were placed into anaerobic containers (AnaeroPack Rectangular Jar) with an appropriate number of microaerophilic environment generating gas packs (AnaeroPack-MicroAero), per manufacturer instructions, and were incubated at 42 °C for 48 ± 1 h. Three uninoculated and untreated chicken wings were also analyzed on each of the inoculation and treatment application days, for natural microflora counts (on Tryptic Soy Agar [Acumedia-Neogen]; 25 °C for 72 h) and for any naturally-present *Campylobacter* populations (on mCCA) on the chicken wings used in the study. The detection limit of the microbiological analysis was 1 CFU/mL.

### 2.5. Statistical Analysis

The study was designed as a 7 (treatments) × 2 (sampling times) factorial for each solution application method (immersion, spraying), blocked by trial day. It was repeated on two separate days, and three samples were analyzed per treatment and sampling time (0 h and 24 h) in each trial (i.e., a total of six samples per treatment and sampling time). For each solution application method, recovered *C. jejuni* populations were statistically analyzed across all treatments within each sampling time (0 h, 24 h), and across the two sampling times for each antimicrobial treatment. Bacterial populations were expressed as least squares means for log CFU/mL of wing rinsate under the assumption of a log-normal distribution of plate counts. Data were analyzed using the emmeans package [21] in R (version 3.5.1). Means were separated with Tukey adjustment using a significance level of α = 0.05.

## 3. Results

### 3.1. Untreated Chicken Wings

Aerobic microbial populations of the uninoculated and untreated chicken wings used for the study ranged from 2.6 to 4.3 log CFU/mL, with a mean of 3.6 ± 0.7 log CFU/mL. Naturally occurring *Campylobacter* populations were not detected (<1 CFU/mL) in five of the six uninoculated and untreated wings analyzed, while the remaining sample had a *Campylobacter* count of 1 CFU/mL. As such, bacterial populations recovered with the mCCA culture medium from inoculated control (untreated) and treated samples (Table 2 and Table 3) were those of the inoculum strains.

Immersion and spray application methods of the test solutions were evaluated on the same experiment day; therefore, the same set of untreated inoculated samples were used as controls for both application methods (Table 2 and Table 3). The inoculation level of *C. jejuni* on the wings following the inoculation procedure, as determined by microbial analysis of untreated inoculated samples, was 3.9 log CFU/mL, and similar pathogen levels were recovered from untreated wings stored aerobically at 4 °C for 24 h (Table 2 and Table 3).

### 3.2. Chicken Wings Treated by Immersion Application of Antimicrobial Treatments

*C. jejuni* populations recovered from immersion-treated wings immediately after treatment (0 h) and after 24 h of refrigerated (4 °C) storage are shown in Table 2. Compared to the untreated control, all six immersion treatments effectively (*p* < 0.05) reduced initial (0 h) inoculated *C. jejuni* populations (3.9 log CFU/mL), with reductions ranging from 0.5 (water) to 2.2 (PAA, and SSS-aPAA) log CFU/mL. Moreover, pathogen counts recovered from wings that had been treated with any of the five tested chemical solutions were 1.2 (SSS) to 1.7 (PAA, SSS-aPAA) log CFU/mL lower (*p* < 0.05) than the pathogen counts of samples that had been treated with water. No (*p* ≥ 0.05) differences in efficacy against *C. jejuni* were observed at the 0-h sampling time between the SSS, formic acid, and FA-aPAA. Additionally, formic acid, PAA, and the two acidified PAA treatments were equally (*p* ≥ 0.05) effective against *C. jejuni* immediately following their application, reducing initial populations by 1.8 (formic acid) to 2.2 (PAA, and SSS-aPAA) log CFU/mL.

Within each immersion treatment, pathogen counts of samples analyzed after the refrigerated storage period were similar (water, PAA; *p* ≥ 0.05) or lower (SSS, formic acid, SSS-aPAA, FA-aPAA; *p* < 0.05) than the counts of corresponding 0-h samples (Table 2). More specifically, at the 24-h sampling time, pathogen counts of wings that had been treated with SSS, formic acid, SSS-aPAA, or FA-aPAA were 0.6, 0.9, 0.8, and >1.2 log CFU/mL lower (*p* < 0.05), respectively, than counts of the corresponding treatments at the 0-h sampling time. Furthermore, it was observed that within the 24-h sampling point, *C. jejuni* counts of wings that had been treated with SSS-aPAA or FA-aPAA were lower (by 0.5 and >0.8 log CFU/mL, respectively; *p* < 0.05) than the counts of samples that had been treated with non-acidified PAA.

### 3.3. Chicken Wings Treated by Spray Application of Antimicrobial Treatments

Results for the spray-treated wings are presented in Table 3. Spray application of the treatments lowered (*p* < 0.05) initial *C. jejuni* populations (3.9 log CFU/mL) by 0.3 (water) to 1.2 (FA-aPAA) log CFU/mL. No (*p* ≥ 0.05) differences in efficacy against the pathogen were noted between the water treatment and SSS treatment. Additionally, formic acid and PAA had similar (*p* ≥ 0.05) immediate (0 h) antimicrobial effects, reducing (*p* < 0.05) initial pathogen populations by 0.7 and 0.9 log CFU/mL, respectively. At the 0-h sampling time, surviving *C. jejuni* populations of wings treated with SSS-aPAA or FA-aPAA were lower (*p* < 0.05) than those of samples treated with SSS or formic acid (by 0.6 and 0.5 log CFU/mL, respectively). No (*p* ≥ 0.05) differences in antimicrobial efficacy were obtained at 0 h between PAA and the two acidified PAA treatments.

*C. jejuni* counts recovered from wings treated with formic acid, SSS-aPAA or FA-aPAA, and stored at 4 °C (24 h), were 0.2, 0.4, and 0.6 log CFU/mL lower (*p* < 0.05), respectively, than the counts obtained for these treatments at the 0-h sampling time (Table 3). However, for samples that had received the water, SSS or PAA treatment, pathogen levels recovered after 24 h of storage were similar (*p* ≥ 0.05) to those obtained immediately after treatment application. Lastly, as seen for the immersion application method (Table 2), although no statistical differences (*p* ≥ 0.05) were observed between the PAA and either of the acidified PAA treatments at the 0-h sampling point, after refrigerated storage, pathogen counts of SSS-aPAA and FA-aPAA spray-treated samples were lower (by 0.4 and 0.7 log CFU/mL, respectively; *p* < 0.05) than those of wings that were spray-treated with PAA (Table 3).

## 4. Discussion

Multiple intervention strategies, including the use of chemical antimicrobial treatments, are used by the U.S. poultry processing industry to reduce the prevalence of *Campylobacter* and *Salmonella* on whole carcasses and parts [11]. These chemical decontamination treatments are applied as sprays and/or immersion (dip) treatments at pre- and post-chill stages of processing [11,22]. In the current study, SSS, formic acid, PAA, and two acidified PAA treatments (SSS-aPAA and FA-aPAA) were evaluated for their antimicrobial effects against *C. jejuni* populations on chicken wings. Overall, all of the chemical treatments were effective (*p* < 0.05) in reducing initial pathogen levels, and under the experimental conditions of the study, greater reductions were obtained when the wings received the treatment by immersion (5 s) than as a spray (4 s) (Table 2 and Table 3).

The antimicrobial effects of SSS against various foodborne pathogens have been previously evaluated, mostly on beef products [23,24,25,26,27,28,29,30] but also on poultry carcasses and parts by a few investigators [18,31,32]. Scott et al. [18] reported a 1.2 log CFU/mL reduction of inoculated (5.5 log CFU/mL) *Salmonella* populations on chicken wings that were immersed for 20 s in a pH 1.1 solution of SSS. In another study [31], immersion of turkey drumsticks in SSS (pH 1.3) for 30 s lowered inoculated (7–8 log CFU/g) *Salmonella* Reading and *Salmonella* Typhimurium populations by 2.2 and 2.4 log CFU/g, respectively. In the current study, *C. jejuni* levels on wings were reduced (*p* < 0.05) by 1.7 and 0.5 log CFU/mL immediately following immersion or spray treatment with SSS (pH 1.2), respectively (Table 2 and Table 3). To our knowledge, there has only been one other published study that has investigated the antimicrobial efficacy of SSS against *Campylobacter* on poultry. In this particular study [32], a 1.5 log CFU/chicken reduction of naturally occurring *Campylobacter* spp. populations was reported when post-chilled whole carcasses were immersed in SSS (pH 1.4) for 15 s.

Published reports on the use of formic acid as a decontamination treatment of poultry are limited. Riedel et al. [33] observed a 1.6 log CFU/mL reduction of *C. jejuni* inoculated on chicken skin that was immersed for 1 min in 2% formic acid. In the present study, the antimicrobial efficacy of 1.5% formic acid against initial populations of *C. jejuni* was similar (*p* ≥ 0.05) to that of SSS, regardless of the application method (Table 2 and Table 3). Specifically, reductions of 1.8 and 0.7 log CFU/mL were obtained for wings immersion- or spray-treated with formic acid, respectively.

As previously mentioned, PAA is currently one of the most commonly used antimicrobials in U.S. poultry slaughter and processing facilities, and its effectiveness in reducing pathogen contamination on poultry-associated products has been extensively reported [11,14,16,18,31,32,34,35,36,37,38,39]. Naturally occurring *Campylobacter* spp. levels were reduced by 2.2 log CFU/chicken when post-chilled whole carcasses were subjected to a 15 s dip in 750 ppm PAA [32]. Nagel et al. [35] also evaluated PAA as a post-chill immersion (20 s) treatment of whole carcasses and reported 1.9 and 2.0 log CFU/mL reductions of inoculated (ca. 5 log CFU/mL) *C. jejuni* populations with 400 ppm and 1000 ppm PAA, respectively. In another study [39], 200 ppm PAA applied as an immersion (60 s) or spray (62 s) treatment lowered *C. jejuni* levels of chicken carcasses by 1.4 and 0.6 log CFU/mL, respectively. PAA was also recently evaluated as a decontamination treatment of skinless, boneless chicken breast fillets [38]. Specifically, breast fillets inoculated with *Campylobacter coli* populations (4.9 log CFU/mL) were reduced by 0.9 and 0.8 log CFU/mL when they were immersed (3.5 L, 4 s) or sprayed (15 mL/s, 5 s) with 500 ppm PAA [38].

While the antimicrobial effects of PAA have been extensively investigated, there are only a few recently published studies on the use of pH-adjusted (acidified) PAA as a decontamination treatment of meat and poultry products [30,31]. In our study, no differences (*p* ≥ 0.05) were obtained between PAA and the acidified PAA treatments (SSS-aPAA and FA-aPAA) with regard to reducing initial (0 h) levels of *C. jejuni* contamination, irrespective of whether the treatments were applied by immersion or in the spray cabinet. Specifically, the three PAA-containing treatments reduced 0 h pathogen populations by 2.1 to 2.2 log CFU/mL in immersion-treated samples, and 0.9 to 1.2 log CFU/mL in spray-treated samples (Table 2 and Table 3). After refrigerated storage (4 °C, 24 h), however, differences (*p* < 0.05) were noted between recovered pathogen populations from wings that had been treated (immersion or spray) with PAA and those that received one of the acidified PAA treatments. While 0 h *C. jejuni* populations of PAA-treated wings remained relatively unchanged (*p* ≥ 0.05) following the 24-h storage period, pathogen levels of 24 h samples that had received either of the acidified PAA treatments were lower (*p* < 0.05; by 0.8 to >1.2 log CFU/mL for immersion-treated samples, and 0.4 to 0.6 log CFU/mL for spray-treated samples) than the populations recovered from the corresponding treatments at 0 h. Acidification of PAA, regardless of the acidifier (i.e., SSS or formic acid), combines two mechanisms of action. PAA is an oxidizing agent that disrupts bacterial cell walls and essential enzyme functions [40,41], and formic acid and SSS cause cytoplasmic acidification which results in the accumulation of protons that leads to the cell using its energy to try to re-establish the intracellular pH [42,43,44]. Therefore, the combination of hurdles of the acidified PAA coupled with the subsequent low-temperature storage conditions probably impeded recovery of sub-lethally injured cells and likely explains the further reduction of *C. jejuni* levels in the 24-h acidified PAA-treated samples. Evidence of sub-lethal cell injury was also observed for wings that were immersed in SSS or formic acid (i.e., without PAA) (Table 2). Scott et al. [18] and Riedel et al. [33] also reported further reductions of pathogen populations following refrigerated storage of SSS- and formic acid-treated samples, respectively.

Two previous studies have evaluated the antimicrobial effects of acidified PAA treatments [30,31]. Similar to the 0 h results of our study, Olson et al. [31] reported no differences between *Salmonella* reductions obtained immediately following treatment (30 s immersion) of turkey drumsticks with 500 ppm PAA or PAA (500 ppm) acidified with SSS (pH 1.3). In contrast to the findings of our study, subsequent storage (4 °C, 24 h) did not result in further reductions of *Salmonella* populations on samples treated with SSS-acidified PAA [31]. Acidified PAA solutions have also been evaluated as spray treatments (10 s, 103 kPa) of prerigor beef carcass surface tissue for reduction of nonpathogenic *Escherichia coli* surrogates for Shiga toxin-producing *E. coli* and *Salmonella* [30]. The authors of this study reported that acidification of PAA (350 ppm or 400 ppm) with 2% acetic acid or pH 1.2 SSS did not (*p* ≥ 0.05) enhance the immediate antimicrobial effects of non-acidified PAA (350 ppm or 400 ppm) [30].

## 5. Conclusions

Results of this investigation demonstrated that all tested chemical interventions (SSS, formic acid, PAA, SSS-aPAA and FA-aPAA) were effective (*p* < 0.05) in reducing *C. jejuni* populations on chicken wings, with greater immediate reductions obtained when the treatments were applied by immersion than by spraying. Acidification of PAA (550 ppm) with pH 1.2 SSS or 1.5% formic acid did not enhance the immediate (0 h) bactericidal effects of non-acidified PAA; however, the combination of hurdles of the acidified PAA treatments and the subsequent chilled storage conditions (4 °C, 24 h) likely prevented recovery of sub-lethally injured bacterial cells. As a result, chicken wings treated with SSS-aPAA or FA-aPAA and stored at 4 °C for 24 h had the lowest pathogen levels. Further research should be conducted to evaluate the efficacy of SSS-aPAA and FA-aPAA in reducing *C. jejuni* contamination on chicken parts when applied under conditions found in commercial processing facilities.

## Figures and Tables

**Table 1:** *Campylobacter jejuni* strains used in the study.

Strain ID	Origin	Source
FSIS21822450	Chicken drumsticks	USDA-FSIS-OPHS ^a^
FSIS21822588	Chicken drumsticks	USDA-FSIS-OPHS
FSIS11815850	Ground chicken	USDA-FSIS-OPHS
CVM N55886	Chicken wings	FDA-CVM ^b^
CVM N56299	Chicken wings	FDA-CVM
CVM N16C024	Chicken breast	FDA-CVM

^a^ U.S. Department of Agriculture, Food Safety and Inspection Service, Office of Public Health Science. ^b^ U.S. Food and Drug Administration, Center for Veterinary Medicine.

**Table 2:** Mean (*n* = 6) *Campylobacter jejuni* populations (log colony-forming units [CFU]/mL ± standard deviation [SD]) for inoculated (six-strain mixture; 3 to 4 log CFU/mL) chicken wings that were left untreated (control) or were immersion-treated (5 s, 500 mL of solution per sample) with various treatment solutions.

Treatment	Mean *C. jejuni* Populations (log CFU/mL ± SD)	
	0 h	24 h
Control	3.9 ± 0.1 ^a,z^	3.7 ± 0.3 ^a,z^
Water	3.4 ± 0.1 ^b,z^	3.2 ± 0.2 ^b,z^
SSS (pH 1.2)	2.2 ± 0.1 ^c,z^	1.6 ± 0.2 ^c,y^
Formic acid (1.5%)	2.1 ± 0.2 ^cd,z^	1.2 ± 0.1 ^cd,y^
PAA (550 ppm)	1.7 ± 0.3 ^d,z^	1.4 ± 0.4 ^c,z^
SSS-aPAA	1.7 ± 0.3 ^d,z^	0.9 ± 0.2 ^de,y^
FA-aPAA	1.8 ± 0.2 ^cd,z^	<0.6 ± 0.5 ^e,y^ *

SSS: sulfuric acid and sodium sulfate blend; PAA: peroxyacetic acid; SSS-aPAA: PAA (550 ppm) acidified with SSS (pH 1.2); FA-aPAA: PAA (550 ppm) acidified with formic acid (1.5%). ^a–e^ Least squares means in the same column without a common superscript letter are different (*p* < 0.05). ^y–z^ Least squares means in the same row without a common superscript letter are different (*p* < 0.05). * One of the six samples analyzed had a *C. jejuni* count that was below the microbial analysis detection limit of 1 CFU/mL; therefore, the mean is reported as < (less than) the mean.

**Table 3:** Mean (*n* = 6) *Campylobacter jejuni* populations (log colony-forming units [CFU]/mL ± standard deviation [SD]) for inoculated (six-strain mixture; 3 to 4 log CFU/mL) chicken wings that were left untreated (control) or were spray-treated (4 s, 69 to 83 kPa) with various treatment solutions.

Treatment	Mean *C. jejuni* Populations (log CFU/mL ± SD)	
	0 h	24 h
Control	3.9 ± 0.1 ^a,z^	3.7 ± 0.3 ^a,y^
Water	3.6 ± 0.1 ^b,z^	3.5 ± 0.2 ^ab,z^
SSS (pH 1.2)	3.4 ± 0.2 ^bc,z^	3.3 ± 0.2 ^bc,z^
Formic acid (1.5%)	3.2 ± 0.2 ^cd,z^	3.0 ± 0.2 ^cd,y^
PAA (550 ppm)	3.0 ± 0.2 ^de,z^	2.8 ± 0.2 ^d,z^
SSS-aPAA	2.8 ± 0.1 ^e,z^	2.4 ± 0.5 ^e,y^
FA-aPAA	2.7 ± 0.1 ^e,z^	2.1 ± 0.4 ^e,y^

SSS: sulfuric acid and sodium sulfate blend; PAA: peroxyacetic acid; SSS-aPAA: PAA (550 ppm) acidified with SSS (pH 1.2); FA-aPAA: PAA (550 ppm) acidified with formic acid (1.5%). ^a–e^ Least squares means in the same column without a common superscript letter are different (*p* < 0.05). ^y–z^ Least squares means in the same row without a common superscript letter are different (*p* < 0.05).

## Data Availability

The data presented in this study are available on request from the corresponding author.
